# How does self-control influences college students’ smartphone dependence: The chain mediating effect of mental toughness and social adaptability

**DOI:** 10.1371/journal.pone.0350219

**Published:** 2026-06-04

**Authors:** Quanwei Shen, Lin Li, Chenglong Li

**Affiliations:** 1 Department of Applied Psychology, Hubei University of Medicine, Shiyan, China; 2 College of Sports Medicine, Wuhan Sports University, Wuhan, China; 3 College of Humanities and Social Sciences, Hubei University of Medicine, Shiyan, China; ISSEP Kef: Universite de Jendouba Institut Superieur du Sport et de l’Education Physique du Kef, TUNISIA

## Abstract

The study aimed to investigate the mediating roles of mental toughness and social adaptability in the relationship between self-control and smartphone dependence among college students. In January 2025, through a combination of convenience sampling and quota sampling methods, a total of 600 college students were selected from Hubei Province for a questionnaire survey. Mediation analysis was conducted with the SPSS PROCESS Model 6 to explore how mental toughness and social adaptability influence the connection between self-control and smartphone dependence. Results indicated that self-control, mental toughness, and social adaptability are all significantly negatively correlated with smartphone dependence. Self-control, mental toughness, and social adaptability show a positive correlation between each pair. Self-control can be associated with smartphone dependence through the path of social adaptability. mental toughness and social adaptability had a chain mediating relationship between self-control and smartphone dependence. The research results suggest that self-control ability is a key related factor for college students’ smartphone dependence. It is necessary for colleges and universities to enhance students’ beliefs and abilities to overcome and solve difficulties, enrich their extracurricular lives, help them better integrate into real society, and use smartphones reasonably.

## 1 Introduction

The “China Internet Development Report 2024” estimates that the number of mobile internet users in China may reach 1,096 billion, constituting 99.7% of the total internet users. This represents an increase of 14.03 million users from December 2023. Furthermore, individuals aged 20–29 comprised 13.1% of the total user base [1]. College students are the primary group of smartphone users and are at high risk for smartphone dependence. More than 25% of college students are troubled by smartphone dependence [2]. Smartphone dependence is a behavioral issue that involves using smartphones excessively or a persistent craving for smartphone use [3].

Contemporary Chinese college students are growing up in the era of mobile Internet and smartphones, and online social media is the key field for college students to build and maintain social relations [4]. When college students face realistic difficulties, such as family expectations, academic setbacks, or peer communication, they tend to seek emotional support and social connection through social platforms like WeChat, QQ, and Weibo [5]. With the help of real-time interaction and a real-time feedback mechanism, online social platforms meet the social needs of college students, while also fostering a significant tendency towards online social dependence among them. When college students are separated from their smartphones, they often feel lonely and anxious, reflecting that their smartphone use behavior has evolved from instrumental needs to emotional dependence [6]. Studies have revealed a connection between smartphone dependence and a range of serious physical and mental health problems among college students. This dependence can lead to insomnia [7], eating disorders [8], persistent shoulder and neck pain [9], as well as increased risks of depression [10] and anxiety [11]. Additionally, smartphone dependence negatively affects their academic performance [12] and interpersonal relationships [13], significantly impacting their daily lives and studies.

In recent years, the phenomenon of smartphone dependence of Chinese college students has attracted wide attention from all walks of life, and formed a multi-level response system composed of education departments, society, universities, families, and individuals, from macro policy to micro intervention. China’s Ministry of Education, Health Commission, and Network Security Informatization Commission successively issued relevant documents to provide a policy basis for mobile phone management and mental health education in schools at all levels [14,15]. Some universities actively carry out a variety of extracurricular activities to provide students with alternative offline life experiences [16]. Some social institutions and media actively advocate the social consensus of rational use of digital products and improve the cognition of college students to use smartphones correctly [17]. Parents can provide stable emotional support for college students by carrying out high-quality communication with their children, creating a healthy and harmonious family atmosphere, and reducing the possibility of college students’ excessive dependence on mobile phones due to stress [18]. Therefore, under the active guidance of all walks of life, it is very necessary to enhance college students’ self-awareness of using smartphones, consciously identify the motivation of using smartphones, monitor the behavior of using smartphones, and pay attention to the emotional reaction of using smartphones, so as to strengthen individual scientific and rational use of smartphones.

According to the theory of ternary interactive decision making, there is a two-way interaction and mutual decision among human behavior, the individual, and the environment [19]. The theory states that there may be a correlation between a person’s abilities and their smartphone-dependent behavior. A large number of studies have shown that self-control is closely related to smartphone dependence and is an important variable to predict smartphone dependence [20,21]. Individuals with higher levels of self-control tended to exhibit lower levels of smartphone dependence [22]. Self-control may be directly or indirectly linked to an individual’s ability to manage smartphone use. Studies have shown that self-control is not only directly related to an individual’s ability to manage smartphone use behavior but may also be associated with smartphone dependence behavior through an indirect path of psychological competence. Specifically, studies have shown that self-control is positively associated with mental toughness, which in turn is associated with lower levels of smartphone dependence [23]. In addition, self-control is positively correlated with social adaptability, including interpersonal communication [24,25], learning ability [26], self-management, and emotion regulation [27–29]. Good social adaptability is associated with lower levels of smartphone dependence [30].

This study seeks to investigate the connections among self-control, mental toughness, social adaptability, and smartphone dependence. By building a model, we aim to explore how mental toughness and social adaptability mediate the relationship between self-control and smartphone dependence. Thus, it provides references and insights for reducing smartphone dependence among college students and promoting their physical and mental health.

### 1.1 Self-control and smartphone dependence

Research shows that college students with greater self-control tend to have lower smartphone dependence compared to those with less self-control [31]. Additionally, higher levels of self-control among college students are associated with lower smartphone dependence [32]. Self-control is a skill that is related to individuals’ ability to resist immediate temptations, manage impulsive behaviors, and pursue actions that align with their long-term goals. This ability is linked to adhering to one’s ideals, values, morals, and societal expectations [33,34]. The dual-system theory suggests that self-control involves two systems: the impulsive system and the control system. Individuals with strong self-control tend to have an effective control system that is associated with managing their impulsive behavior [35]. A study emphasizes that impulsive smartphone usage, often triggered by environmental cues, is positively related to an individual’s dependence on smartphones [36]. Students with strong self-control generally possess more robust control systems, which are associated with effectively suppressing impulsive smartphone use behaviors and lower levels of dependence. In contrast, individuals with lower self-control tend to have weaker control systems and greater difficulty in suppressing impulsive smartphone use behaviors, which are related to higher levels of dependence. Therefore, we propose the following research hypothesis: H1: Self-control has a significant negative correlation with smartphone dependence.

### 1.2 The mediating role of mental toughness

Mental toughness refers to the dynamic process by which individuals achieve positive adaptation in the face of significant adversity or significant stressors [37]. There may be a complex bidirectional association between self-control and mental toughness. Individuals with high psychological mental toughness tend to show stronger self-control [38], and self-control training is also associated with improved psychological mental toughness [39]. Self-control is generally regarded as a more fundamental trait resource that is related to individuals’ ability to maintain goal-directed behavior in the face of temptation, stress, and interference [40]. This ability is linked to the cognitive and behavioral aspects that are associated with the development of psychological mental toughness. Individuals with high self-control are more likely to be associated with positive coping strategies that help maintain or restore psychological balance in adversity [41]. Therefore, based on theoretical considerations, self-control was treated as a predictor variable of mental toughness in this study.

Research has shown that self-control is positively associated with levels of mental toughness [21,42]. In a study focused on college students, it was found that those with higher self-control tended to exhibit greater mental toughness compared to those with lower self-control [43]. Mental toughness is an internal capacity that allows individuals to utilize protective resources for harmonious development and effective adaptation in the face of setbacks [37,44]. Kumpfer’s toughness framework model highlights a viewpoint: when one’s stable internal status is affected by external factors, a new stable state emerges through the interaction of protective and negative factors. When the protective factor has a greater impact than the negative factor, individuals may achieve a higher level of toughness than before, which is associated with enhanced mental toughness. Conversely, if risk factors dominate, individuals may develop a lower level of mental toughness, which is related to diminished psychological strength [45]. Higher self-control is considered a protective factor in relation to mental toughness. Individuals with strong self-control tend to possess effective problem-solving abilities and skills, which are associated with better mobilization of their own resources and utilization of external environmental resources to manage stress and challenges, and this in turn is related to higher levels of mental toughness [46].

On the other hand, mental toughness is negatively associated with smartphone dependence [47]. The compensatory Internet use model posits that people tend to seek psychological comfort in the Internet world as a response to social problems, negative emotions, and unmet needs in their real lives [48]. Individuals with low mental toughness tend to experience more negative emotions when faced with difficulties and challenges. If these negative emotions are not effectively managed, they may turn to their smartphones to escape from reality, and this pattern is related to smartphone dependence [49]. Research indicated that college students with high mental toughness exhibited a relatively lower degree of dependence compared to their peers with lower mental toughness [50]. Thus, we propose the following research hypothesis: H2: Mental toughness plays a significant mediating role in the relationship between self-control and smartphone dependence.

### 1.3 The mediating role of social adaptability

Social adaptability is the ability to skillfully adjust to one’s social environment, cultivate harmonious interpersonal relationships, and improve social skills, problem-solving capabilities, and interaction techniques [51]. Research indicates that self-control is closely linked to different aspects of social adaptability, such as interpersonal communication, learning ability, self-management, and adherence to social norms. People who have stronger self-control tend to respond to situations more rationally, demonstrate greater empathy, and generally possess enhanced interpersonal skills [24,25]. This pattern is associated with increased prosocial behaviors and adherence to social norms. People with stronger self-control are more effective at regulating their attention, which is related to the ability to allocate cognitive resources efficiently and complete academic tasks successfully [26]. Furthermore, those with a high level of self-control are likely to excel in regulating their emotions. According to emotion regulation theory, individuals cope with stress or negative emotions by actively adjusting their emotional experiences, physiological reactions, and behavioral strategies [52]. People with higher self-control tend to effectively employ emotional regulation techniques to handle the emotional fluctuations caused by external stimuli [53]. Self-control is associated with reduced procrastination and aggression and more regulated actions [27–29]. This ability is related to maintaining a stable emotional state and is associated with enhanced adaptability in social situations [54].

At the same time, there may be a close link between social adaptability and smartphone dependence. Research shows that college students with low social adaptability tend to demonstrate higher levels of smartphone dependence compared to those with strong social adaptability [11,30]. The compensatory internet use theory suggests that when individuals’ basic psychological needs are unmet in reality, they may seek psychological comfort on the Internet as a form of compensation [48]. This mechanism is related to individuals’ tendency to escape real life and become engaged with the Internet. Good social adaptability is associated with individuals’ ability to cope with pressures and meet their basic psychological needs in real life. When these needs are fulfilled, students are less likely to rely on the online world for comfort. Conversely, college students with low social adaptability may face more academic challenges and social anxiety due to insufficient social skills, a lack of coping strategies, and weak interpersonal relationships. When confronted with stress and difficulties, they may feel a lack of competence in real life. Consequently, to fulfill their unmet psychological needs, they often pursue virtual achievements online through smartphones, and this pattern is related to increased smartphone dependence [55]. Thus, we propose the following research hypothesis: H3: Social adaptability plays a significant mediating role in the relationship between self-control and smartphone dependence.

### 1.4 The chained mediating role of mental toughness and social adaptability

Empirical studies indicate a strong association between psychological mental toughness and social adaptability [56,57]. While both concepts involve adaptation, they differ in their core focus. Psychological mental toughness emphasizes internal psychological resources and coping processes during stressful situations, which primarily reflect active resistance to stress [37]. In contrast, social adaptability pertains to external behaviors exhibited by individuals within typical social environments, highlighting active integration [58]. Based on this conceptual distinction, mental toughness can be viewed as an implicit psychological resource, whereas social adaptability can be regarded as an explicit behavioral skill. A study indicates that mental toughness is closely associated with social adaptability and is related to individuals’ ability to adjust socially [59]. According to Richardson’s toughness process model, when individuals face external stressors, their toughness is associated with internal factors that help them seek social, cultural, and material resources to manage stress and challenges. This process is related to a well-adapted developmental state [60]. Individuals with higher self-control tend to have higher levels of mental toughness [61], and they are more likely to take an optimistic and positive approach when facing difficulties and challenges [62]. These individuals effectively use various strategies to address setbacks, which is associated with their ability to integrate quickly into social environments. In contrast, those with lower self-control often show poor stress resistance and struggle with problem-solving, and this pattern is related to decreased toughness. This, in turn, is associated with lower social adaptability, making it more challenging for them to manage the stresses in reality [63]. The compensatory internet use theory shows that smartphone dependence among college students is closely related to negative emotions arising from social maladjustment [48]. College students with low social adaptability tend to resort to mobile internet usage to alleviate the negative emotions associated with their social challenges, and this pattern is related to smartphone dependence. Thus, the level of self-control among college students may be associated with their social adaptability through mental toughness, which in turn is related to their level of smartphone dependence. Therefore, we propose the following research hypothesis: H4: Mental toughness and social adaptability jointly act as a chained mediator in the statistical relationship between self-control and smartphone dependence.

**Fig 1:**
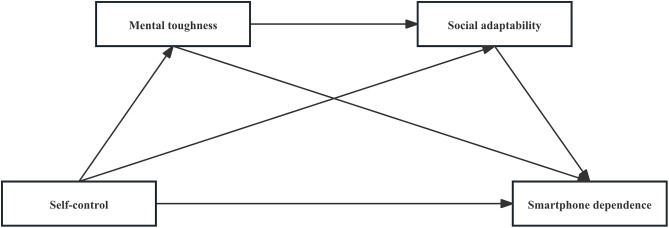
Hypothetical model diagram.

Based on the theoretical analysis and research hypothesis, this study’s hypothesis model is constructed, as illustrated in Fig 1.

## 2 Materials and methods

### 2.1 Participants and procedures

Approval for this study was granted by the Biomedical Ethics Committee of Hubei University of Medicine (2024-RE-051), and it was conducted in accordance with relevant ethical guidelines. The research was conducted at four universities in Hubei Province from January 3rd to 25th, 2025. 150 college students were selected from each university, with a total of 600 students. Prior to conducting the questionnaire, the researchers communicated with the heads of the four universities. A combination of convenience sampling and quota sampling was used for sampling in this study. The details are as follows: (1) Quota sampling: In this study, the quota is based on the ratio of 1∶1 in terms of gender and urban and rural household registration, and the quota is based on the ratio of 1∶1∶1∶1 in terms of freshman, sophomore, junior, and senior years. (2) Convenience sampling: according to the pre-set quota conditions, temporary recruitment points were set up in the canteen, library, and other densely populated areas for convenience sampling. The first step was on-site recruitment and oral informed consent. Participants were given a uniform verbal explanation by the researcher on site about the purpose of the study, anonymity, the principle of voluntary participation, and the right to withdraw at any time to ensure that participants were fully informed about the study information. After obtaining the participants’ verbal consent to participate in the investigation, we also required them to read the paper-based informed consent form. The second step was to fill out the online questionnaire. After the oral instructions, students voluntarily used their personal mobile phones to enter the online questionnaire platform (Questionnaire Star) to fill in the questionnaire. The informed consent statement was again presented in text on the homepage of the questionnaire, and participants clicked “Agree and begin to answer” to enter the formal questionnaire. In this study, subjects were recruited according to the quota target as much as possible during the recruitment process. However, in the process of filling out the questionnaire, male college students are lower than female college students, and senior students are lower than junior students. In the process of questionnaire collection and data processing, the quality of questionnaire filling also showed the same trend. The collection of 490 valid questionnaires, and the effective response rate was 81.67%, with 198 male participants (40.41%) and 292 female participants (59.59%). The participants, on average, were 19.27 years old, with a standard deviation of 1.08 years. The breakdown of students by year is as follows: 136 were freshmen (27.76%), 131 were sophomores (26.73%), 124 were juniors (25.31%), and 99 were seniors (20.20%). Additionally, 206 participants (42.04%) were only children, while 284 (57.96%) had siblings. Regarding their locations, 270 participants (55.10%) were from urban areas, and 220 participants (44.90%) were from rural areas.

### 2.2 Research method

#### 2.2.1 Self-control scale.

In this study, the self-control scale of college students compiled by Tan Shuhua was used to measure the self-control level of college students. The scale included 5 dimensions: impulse control, healthy habits, resistance to temptation, work concentration, and moderate entertainment, a total of 19 questions. The scale is scored on a 5-point scale, with 1 representing “completely inconsistent” and 5 representing “completely consistent”. In terms of the reliability of the scale, the Cronbach α coefficient was 0.862. In terms of validity, X2/df = 1.533, RMSEA = 0.050, GFI = 0.91, IFI = 0.93, NNFI = 0.91, CFI = 0.93 [64], indicating that the scale had good reliability and validity. This scale has been widely used to evaluate the self-control level of Chinese college students [65–67]. In this study, item scores were summed and averaged, with higher scores indicating a higher level of self-control among college students. The Cronbach α coefficient of the scale in this study was 0.85.

#### 2.2.2 Connor-Davidson Resilience Scale (Chinese version).

The Chinese version of the Connor-Davidson Resilience Scale was used to measure the mental toughness of college students. The scale included 3 dimensions: optimism, strength, and resilience, with a total of 25 items. The scale was scored on a 5-point scale, with 0 indicating “never” and 4 indicating “always”. The Cronbach α coefficient of the scale was 0.91 [68]. In terms of the validity of the scale, X2/df = 2.685, CFI = 0.935, TLI = 0.928, RMSEA = 0.060, RMR = 0.037 [69], indicating that the scale has good reliability and validity. The scale has been widely used to evaluate the level of mental toughness of Chinese college students [70–72]. In this study, item scores were summed and averaged, with higher scores indicating higher levels of mental toughness among college students. The Cronbach α coefficient of the scale in this study was 0.90.

#### 2.2.3 Social adaptability diagnostic scale.

In this study, the social adaptability scale developed by Zheng Richang was used to measure the social adaptability of college students [73]. The scale included 5 dimensions: self-management, willingness to express, peer relationship, learning ability, and compliance, with a total of 20 items. A 3-point scoring system was used: for odd items, “yes” corresponded to −2 points, “uncertain” corresponded to 0 points, and “no” corresponded to 2 points. For even items, “no” corresponds to −2 points, “uncertain” to 0 points, and “yes” to 2 points. The Cronbach α coefficient of the scale was 0.937. In terms of validity, X2/df = 3.125, RMSEA = 0.043, GFI = 0.849, NFI = 0.98, CFI = 0.923, TLI = 0.876 [74]. It shows that the scale has good reliability and validity. The scale has been widely used to evaluate the social adaptability of Chinese college students [75,76]. After the odd item scores were reversed in this study, all item scores were summed to obtain a total score, with higher total scores indicating greater social adaptability. The Cronbach α coefficient of the scale in this study was 0.76.

#### 2.2.4 Smartphone addiction scale for adults.

In this study, the Adult Smartphone Addiction Scale developed by Chen Huan was used to measure the level of smartphone addiction in college students. The scale consists of 6 dimensions: app usage, app updates, withdrawal reactions, salience, social functioning impairment, and physical discomfort, as well as 20 items. The scale is scored on a 5-point scale, with 1 representing “completely inconsistent” and 5 representing “completely consistent”. The Cronbach α coefficient of the scale was 0.909. In terms of the validity of the scale, X2/df = 2.13, RMSEA = 0.043, GFI = 0.94, IFI = 0.94, SRMR = 0.000, CFI = 0.94 [77], indicating that the scale has good reliability and validity. This scale has been used to assess the level of smartphone addiction among Chinese adolescents [78]. In this study, item scores were summed and averaged, with higher scores indicating higher levels of smartphone addiction among college students. The Cronbach α coefficient of the scale in this study was 0.90.

## 3 Results

### 3.1 Common method deviation test

The Harman single factor test was utilized to assess common method deviation. Unrotated exploratory factor analysis extracted 26 factors with eigenvalues above 1. A single factor accounted for a maximum of 17.71% of the variance, which is less than the 40% threshold. The Full Collinearity Test was used to determine the severity of common method bias. The results showed that the Inner VIF values of all latent variables in this study ranged from 1.000 to 2.006, which was well below the suggested threshold of 3.3. Thus, this research does not demonstrate a notable common method bias.

### 3.2 Descriptive statistics and correlation analysis

The main variables’ correlation analysis outcomes are presented in Table 1. Self-control scores are significantly positively correlated with mental toughness and social adaptability scores, and significantly negatively correlated with smartphone dependence scores. The mental toughness score was positively correlated with the social adaptability score and negatively correlated with the smartphone dependence score. The social adaptability score was notably negatively correlated with the smartphone dependence score.

**Table 1:** Correlations among variables.

Variable	M	SD	1	2	3	4
1. SC	3.41	0.49	1			
2. MT	2.22	0.38	0.52**	1		
3. SA	0.08	0.64	0.46**	0.59**	1	
4. SPD	2.88	0.56	–0.59**	–0.34**	–0.45**	1

Note: M indicates mean; SD indicates standard deviation; SC indicates self-control; MT indicates mental toughness; SA indicates social adaptability; SPD indicates smartphone dependence; ** indicates p < 0.01.

### 3.3 Analysis of intermediary effect

To eliminate the influence of different scale scoring methods and the number of items on the comparison of effect sizes, z-scores were standardized for all predictor and outcome variables before regression analysis (mediation effect analysis). The chain mediation test was performed using Model 6 in the PROCESS 3.5 SPSS plug-in, with the findings presented in Table 2. The score for self-control was significantly negatively associated with the score for smartphone dependence (β = -0.59, p < 0.001). There was a significant positive association between self-control score and mental toughness score (β = 0.52, p < 0.001). A significant positive association was found between self-control score and social adaptability score (β = 0.20, p < 0.001), and the mental toughness score also showed a notable positive association with the social adaptability score (β = 0.48, p < 0.001). With smartphone dependence as the dependent variable and self-control, mental toughness, and social adaptability as the independent variables, there was a significant negative association between self-control scores and smartphone dependence (β = -0.52, p < 0.001) and between social adaptability scores and smartphone dependence (β = -0.26, p < 0.001), but mental toughness was not significantly associated with smartphone dependence (β = 0.09, p > 0.05).

**Table 2:** Mediation model test.

Outcome variable	Predictor variable	β	S.E.	t	R2	F
SPD	SC	−0.59	0.04	−16.23***	0.35	88.93***
	Gender	0.03	0.07	0.46		
	Age	−0.01	0.04	−0.02		
MT	SC	0.52	0.04	13.49***	0.23	49.22***
	Gender	−0.06	0.08	−1.67		
	Age	0.07	0.04	1.74		
SA	SC	0.20	0.04	4.77***	0.38	73.90***
	MT	0.48	0.04	11.42***		
	Gender	−0.02	0.07	−0.63		
	Age	−0.02	0.04	−0.62		
SPD	SC	−0.52	0.04	˗12.29***	0.40	64.09***
	MT	0.09	0.05	1.93		
	SA	−0.26	0.04	−5.88***		
	Gender	0.01	0.07	0.24		
	Age	−0.01	0.04	−0.11		

Note: *** indicates p < 0.001; * indicates p < 0.05; SC indicates self-control; MT indicates mental toughness; SA indicates social adaptability; SPA indicates smartphone dependence.

We made VIF diagnoses for the predictor variables in the regression model. The results showed that VIF = 1.64 for self-control, VIF = 2.01 for mental toughness, and VIF = 1.77 for social adaptability, all of which were far below the commonly used critical value of 5. Therefore, there is no multicollinearity problem with the mediation model in this study.

**Fig 2:**
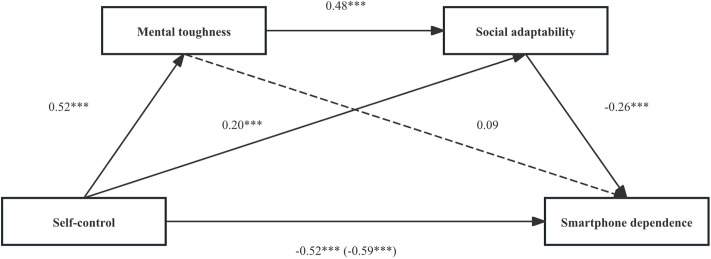
The chained mediation model (*** indicates p < 0.001).

A deviation-corrected percentile Bootstrap method (repeated sampling 5000 times) was used to further examine the mediating effect. Table 3 indicates that the total association between self-control and smartphone dependence (standardized coefficient) was -0.59. After considering mental toughness and social adaptability, the direct association between self-control and smartphone dependence was -0.52 (accounting for 89.39% of the total association), and the confidence interval did not contain 0, indicating that the direct association was significant. The indirect path association of “self-control → mental toughness → smartphone dependence” was 0.05 (accounting for 7.58% of the total association), and the confidence interval contained 0, indicating that the indirect association was not significant. The indirect path association of “self-control → social adaptability → smartphone dependence” was -0.05 (accounting for 7.58% of the total association), and the confidence interval did not contain 0, indicating that the indirect association was significant. The indirect path association of “self-control → mental toughness → social adaptability → smartphone dependence” was -0.07 (accounting for 10.61% of the total association), and the confidence interval did not contain 0, indicating that the indirect association was significant. Fig 2 illustrates the chain-mediation model.

**Table 3:** Mediation model path analysis.

Trails	Estimate	S.E.	Proportion	95% Confidence Interval
SC → SPD	−0.59	0.05	89.39%	[-0.76，-0.60]
SC → MT → SPD	0.05	0.02	7.58%	[-0.01，0.10]
SC → SA → SPD	−0.05	0.02	7.58%	[-0.09，-0.03]
SC → MT → SA → SPD	−0.07	0.02	10.61%	[-0.10，-0.04]
Total effect	−0.66	0.04	100%	[-0.60，-0.45]

Note: SC indicates self-control; MT indicates mental toughness; SA indicates social adaptability; SPD indicates smartphone dependence.

The direct association between self-control and smartphone dependence (β = -0.59, accounting for 89.39% of the total association) was much larger than the indirect associations. Although the simple mediation of social adaptability and the chain mediation path of mental toughness and social adaptability were statistically significant, the actual amount of variation explained was very limited. Meanwhile, the results showed that “the indirect association of self-control → mental toughness → smartphone dependence did not reach statistical significance, but the effect size was positive (β = 0.05).” Combined with the mediation model test data in Table 2, it was found that although the path coefficient between mental toughness and smartphone dependence was not significant, the path coefficient showed a positive sign. This trend may suggest that psychological mental toughness plays a complex role in the relationship between self-control and smartphone dependence. As this pathway is not significant, it is not discussed further in this study.

## 4 Discussion

### 4.1 Self-control and smartphone dependence

This study reveals a negative correlation between self-control and smartphone dependence. Higher levels of self-control are associated with lower levels of smartphone dependence. This supports the research hypothesis H1 and is consistent with findings from earlier studies [20,21]. Self-control is the most basic and important factor related to college students’ smartphone dependence. In terms of effect size, self-control had the largest effect size among all variables. The total association between self-control and smartphone dependence included direct and indirect associations. Self-control was not only directly associated with lower smartphone dependence, but also showed indirect associations through the chain mediating path of psychological mental toughness and social adaptability.

According to the two-system theory of self-control, when the control system is strong, it is associated with effective use of cognitive processing and attention resources to evaluate, monitor, and manage impulsive behaviors and automatic reactions, thereby being related to enhanced regulatory ability of the impulse system [35]. College students with a high level of self-control tend to avoid temptations, limit entertainment, and consciously avoid distractions from internet notifications while using their phones [79]. This self-regulation is associated with reduced excessive dependence on smartphone entertainment features. Conversely, lower self-control is associated with weaker impulse control, which is related to an increased urge to use smartphones for immediate gratification. Consequently, this is associated with a significant rise in both the duration and frequency of smartphone usage [80]. People with lower self-control tend to spend more time on their phones and use them more frequently to satisfy immediate impulses, and this pattern is related to issues such as internet dependence [81,82] and smartphone dependence [83].

### 4.2 Mediating role of mental toughness

The study results showed that mental toughness did not play a significant mediating role in the relationship between self-control and smartphone dependence. Therefore, the research hypothesis H2 was not supported. Through testing the mediation model, it was found that the primary reason for the lack of significant mediation was the non-significant correlation between toughness and smartphone dependence. Previous studies have shown that mental toughness is negatively correlated with smartphone dependence [47,49]. Meanwhile, other studies have shown that the correlation between mental toughness and smartphone dependence is not significant [84]. This suggests that the direct association between mental toughness and smartphone dependence remains unclear, which may explain why the mediating role of mental toughness in the relationship between self-control and smartphone dependence is not significant. Further research is needed to examine the association between mental toughness and smartphone dependence.

### 4.3 Mediating role of social adaptability

The study results showed that social adaptability among college students plays a significant mediating role in the relationship between self-control and smartphone dependence, thereby supporting research hypothesis H3. This outcome is consistent with previous research, which suggests that higher self-control is associated with better social adaptability [46,85]. Higher levels of social adaptability among college students are related to lower levels of smartphone dependence [86]. According to the theory of emotion regulation, individuals actively adjust their emotional experiences, physiological responses, and behavioral strategies to cope with stress or negative emotions [52], and this process is related to enhanced adaptability in social situations [54]. Effective self-control is associated with college students’ ability to allocate cognitive resources efficiently, manage themselves better, and integrate more successfully into their lives, studies, and interpersonal relationships, and these factors are in turn related to better social adaptability [87,88]. Good social adaptability has been associated with improved life satisfaction [89], better mental health [90], and a smoother transition into real-life situations. Meanwhile, people who lack self-control tend to be more impulsive [91]. They are more likely to violate social norms and behave impulsively in communication and behavior, which is related to poor interpersonal relationships and difficulties in social adaptation [46]. College students with lower social adaptability tend to face more challenges with negative emotions in real life compared to those with higher social adaptability [30]. The compensatory internet use theory posits that experiencing major negative emotions is associated with the development of smartphone dependence behaviors [48]. Therefore, college students with poor social adaptability may experience more negative emotions in real life and may turn to their smartphones to alleviate these feelings, and this pattern is related to more frequent and longer smartphone use, which in turn is associated with dependency behaviors [30,92].

### 4.4 The chain mediating effect of mental toughness and social adaptability

This study’s findings suggest that college students’ mental toughness and social adaptability play a mediating role in the relationship between self-control and smartphone dependence. Therefore, the research hypothesis H4 was supported. College students with strong self-control tend to handle their emotions and behaviors when facing pressure and challenges, and they are associated with strong willpower, confidence, and high mental toughness [21,43,61]. Higher mental toughness is related to better coping with social pressure and subsequently to better social adaptability [59,63]. Conversely, college students with low self-control tend to show higher impulsiveness in behavior and emotion, which is associated with psychological collapse and giving up when facing pressure and challenges in life, and they often have low mental toughness [61,93]. Low mental toughness is associated with difficulties in coping with real-life pressures and challenges, and this is related to low social adaptability.

According to the compensatory use theory of Internet dependence, the formation of individual smartphone dependence is related to real-life emotional experiences [48]. College students with good social adjustment tend to have higher positive emotions, such as life satisfaction [89,94], happiness [95], and a sense of belonging [96]. When college students experience more positive emotions in real life, this is related to active engagement with reality and less overindulgence in the online world, and they are associated with lower dependence on their smartphones [30]. Negative emotions are more likely to be experienced by college students with poor social adaptation [97] such as low self-esteem [98], anxiety [99], and stress [100], in real life. To alleviate the discomfort caused by negative emotions, college students tend to use the mobile Internet to seek pleasure and euphoria. However, the pleasure and euphoria obtained through the mobile Internet are short-lived, and the negative emotions associated with social maladjustment tend to reappear when Internet use ceases. In order to escape the persistent influence of negative emotions, college students tend to use the Internet on their smartphones more frequently and for longer periods.

### 4.5 Implications and limitations

The results of this study suggest several implications for the prevention and management of smartphone dependence among college students: (1) Self-control is closely related to smartphone dependence in college students. Therefore, efforts to enhance self-control regarding smartphone use may be associated with reduced frequency of use, better management of usage urges, and improved behavioral control. College counselors and teachers may help college students identify their motivations for smartphone use, which is associated with less aimless use. College students are encouraged to engage in self-monitoring and mutual monitoring of mobile phone use time, and to set a daily limit on mobile phone use time. At the same time, the academic affairs office of the university may consider setting up smartphone-free classrooms in study rooms, libraries, and other learning places; such environmental modifications are associated with reduced temptation to use smartphones. (2) Social adaptability plays a mediating role in the relationship between self-control and smartphone dependence. Therefore, university mental health education centers may consider conducting group counseling for college students with poor social adaptability, as this is associated with improved interpersonal communication, conflict resolution, and rule compliance skills. Enhancing college students’ belief and ability to overcome difficulties is related to better integration into campus life. Secondly, the student affairs department of the university may enrich the extracurricular life of college students and encourage participation in offline activities, which is associated with experiencing social enjoyment in real interactions and replacing virtual communication with real interactions. (3) mental toughness did not play a significant mediating role in the relationship between self-control and smartphone dependence, but it did play a role in linking self-control and social adaptation within the chain mediation. Therefore, improving psychological mental toughness through self-control, which is associated with helping college students better adapt to real life, may be relevant for reducing college students’ smartphone dependence.

Limitations of this study: (1) This study used a combination of quota sampling and convenience sampling. Although the quota targets were set in terms of gender, grade, and urban and rural areas, and dynamic adjustment was made in the process of data collection, it did not meet the representative requirements of strict probability sampling, and the results should be promoted with caution. Among them, male college students are not willing to fill in the questionnaire, the proportion of male college students in the invalid questionnaires is high, and the proportion of female college students in the final sample is high, which may have a certain impact on the conclusion of the study. Future studies can adopt targeted recruitment or increase the duration of recruitment to ensure a balanced gender ratio. Secondly, this study only selected four universities in Hubei Province for sampling, in which the proportion of only children is higher than the average level of the national college students, which may be due to the high proportion of Hubei college students in the sample and related to the implementation background of the fertility policy in Hubei Province. The above limitations lead to the conclusion that one should be more cautious in further generalization. In the future, stratified random sampling or a multi-center large sample survey can be used to further verify the conclusions of this study in different regions and different family structures. (2) This was a cross-sectional study. Although an association between self-control and smartphone dependence can be demonstrated to some extent, as well as the mediating role of psychological mental toughness and social adaptability, clarifying causality is challenging. In particular, the relationships among self-control, mental toughness, and smartphone dependence deserve further study. There may be a bidirectional reinforcement mechanism between mental toughness and self-control: individuals with high mental toughness may be more likely to develop self-control. and individuals with strong self-control may be more likely to remain resilient in the face of adversity. Future studies could use longitudinal tracking designs or experimental studies to further examine the dynamic causal relationship between these variables. Second, it is necessary to further explore the relationship between sub-dimensions of mental toughness and mobile phone dependence and to examine the differential role of mental toughness in different contexts. At the same time, other potential mediating variables can be introduced to more fully reveal the multiple pathways through which self-control affects mobile phone dependence. (3) This study used self-report questionnaires to collect data, which may lead to social expectation and fatigue effects. In subsequent studies, it is crucial to obtain data on variables in different ways. The study only focused on the intrinsic personal factors of smartphone dependence. But external factors, such as school and family, can lead to smartphone dependence. In the subsequent research design, the study must consider external factors to further explore how internal and external factors are related to smartphone dependence among college students. (4) The four scales used in this study used different scoring methods. Although we unified the dimensions through z-score standardization and took quality control measures such as double-check and automatic calculation, different scoring methods may still increase the operational risk of data processing. Future studies may consider using a unified scoring format to design the scale, thereby further reducing the possibility of scoring errors.

## 5 Conclusions

This study examined the relationships between self-control, mental toughness, social adaptation, and mobile phone dependence in college students. There is a significant positive correlation between self-control and smartphone dependence, and self-control is a key related factor of smartphone dependence among college students. It is necessary to strengthen the self-control ability of college students, reduce the time and frequency of smartphone use, and standardize their smartphone use behavior. Second, self-control can be associated with smartphone dependence through the path of social adaptability. It is necessary to enhance college students’ social adaptability, enrich their extracurricular lives, and guide them in using smartphones reasonably and effectively. Finally, mental toughness and social adaptability had a chain mediating relationship between self-control and smartphone dependence. It is necessary to enhance students’ belief and ability to overcome and solve difficulties, helping them better integrate into real society and break their dependence on virtual life.

## Supporting information

S1 Data Data used in analysis. (XLSX)
